# Partial Purification, Identification, and Quantitation of Antioxidants from Wild Rice (*Zizania latifolia*)

**DOI:** 10.3390/molecules23112782

**Published:** 2018-10-26

**Authors:** Mei-Jun Chu, Xin-Min Liu, Ning Yan, Feng-Zhong Wang, Yong-Mei Du, Zhong-Feng Zhang

**Affiliations:** 1 Tobacco Research Institute, Chinese Academy of Agricultural Sciences, Qingdao 266101, China; chumjun@163.com (M.-J.C.); liuxinmin@caas.cn (X.-M.L.); yanning@caas.cn (N.Y.); 2 Institute of Food Science and Technology, Chinese Academy of Agricultural Sciences, Beijing 100193, China; wangfengzhong@caas.cn

**Keywords:** wild rice, antioxidant, macroporous resins, LC-MS/MS, phenolics, procyanidins

## Abstract

To provide further insights into the potential health-promoting antioxidants from wild rice (*Zizania latifolia*), which is an abundant but underutilized whole grain resource in East Asia, a partial purification based on D101 macroporous resin was carried out for the purification and enrichment of the antioxidants from the bioactive ethanol extracts of wild rice. On that basis, 34 phenolic compounds in the antioxidant fractions were identified by a high-performance liquid chromatography-linear ion trap quadrupole-Orbitrap-mass spectrometry (HPLC-LTQ-Orbitrap-MS*^n^*). The results suggested that phenolic acids could be enriched in the 10% ethanol-eluted fraction whereas flavonoids (including procyanidins and flavonoid glycosides) could be enriched in 20–30% ethanol-eluted fractions. A quantitative analysis determined by the multiple reaction monitoring mode of the ultra-performance liquid chromatography-triple quadrupole-tandem mass spectrometry (UPLC-QqQ-MS/MS) revealed a high content of procyanidins in wild rice. Compared with phenolic acids, flavonoids may contribute more to the potent antioxidant activity of wild rice. This is the first study on the antioxidants from wild rice *Z. latifolia.* These findings provide novel information on the functional components of wild rice, and will be of value to further research and development on *Z. latifolia*.

## 1. Introduction

Wild rice is the seed of an aquatic plant belonging to the genus *Zizania*, family Poaceae. Among the four species of genus *Zizania* around the world, *Z. aquatica*, *Z. palustris*, and *Z. texana* are indigenous to North America, whereas *Z. latifolia* is native to East Asia [1]. In China, *Z. latifolia* is widely distributed in areas along the Yangtze and Huai Rivers without any cultivation and domestication [2]. Wild rice is an age-old grain that has been used to treat diabetes and other diseases associated with nutrition, in Chinese medicinal practice, with a recorded history of over three thousand years of use in China. Today its use as a grain has almost disappeared, owing to the very different ripening times and easy seed shattering of the cereal [3,4,5]. In North America, dehulled but unpolished wild rice was historically consumed by Native Americans as a staple food [6]. Since the late 20th century, a growing commercialization of wild rice has been emerged to meet the increased demand for health-promoting cereals. In recent years, North American wild rice has been widely used in gourmet food products because of its unique flavor, color, and texture [7].

With its nutritional quality characterized by a high content of proteins, dietary fiber, minerals, vitamins, and other bioactive phytochemicals (such as phenolics and γ-oryzanols), and a low fat content, wild rice was recognized as a whole grain by the U.S. Food and Drug Administration (FDA) in 2006 [4,7,8,9]. Epidemiological studies have demonstrated that the regular consumption of whole grains is beneficial to human health and can reduce the risk of non-communicable diseases such as obesity, diabetes, and cardiovascular diseases [10,11].

Most of the reports on wild rice have focused on its nutrients and health benefits [4,12]. Phytochemicals are key contributors to the health benefits of whole grains, due to their bioactivities, especially antioxidant capacities [10]. Phytochemicals in wild rice have been investigated to a much lesser degree in comparison with those in other cereal grains. To date, there have been only three reports on the characterization of antioxidants from wild rice. Specifically, Qiu et al. identified eight soluble and insoluble monomeric phenolic acids, four ferulate dehydrodimers, and two sinapate dehydrodimers from wild rice *Z. aquatica* by HPLC-MS/MS [7]. Fourteen phenolic acids and six flavonoids (including catechin, epicatechin, epigallocatechin, rutin, quercetin, and kaempferol) in free and bound phenolic fractions of wild rice *Z. aquatica* were determined using HPLC, by Sumczynski et al. [8]. Moreover, Qiu et al. identified three flavonoid glycosides (including diglucosyl apigenin, glucosyl-arabinosyl apigenin, and diarabinosyl apigenin) and six flavan-3-ols (including catechin, epicatechin, and four oligomeric procyanidins) from wild rice *Z. palustris* and *Z. aquatica*, via HPLC-MS/MS [13]. Earlier studies have shown that the other wild rice species *Z. latifolia*, native to East Asia, has a high nutritional value [14,15], and was effective in suppressing hyperlipidemia and oxidative stress, preventing obesity and liver lipotoxicity, and alleviating insulin resistance induced by a high-fat/cholesterol diet in rats [3,5,16]. However, no investigation on the antioxidant activities and bioactive compounds from East Asian wild rice *Z. latifolia* has been reported.

It is well-known that unpurified crude plant extracts always contain carbohydrates, proteins, and other impurities, which may limit further identification and even the application of the bioactive substances [13,17]. Therefore, it is of great importance to purify antioxidants from wild rice. Purification of phytochemicals from wild rice has been little studied apart from one preliminary report by Qiu et al., who fractionated crude extracts of North American wild rice on a Sephadex LH-20 column to improve the detection of procyanidins [13]. As an efficient and practical adsorption material, macroporous resins have been widely used in the purification and separation of phytochemicals, for their many advantages, including suitable adsorption and desorption capacities, high adsorption selectivity, low cost, easy recycling, lower pollution, and suitability for large-scale production [18]. Nevertheless, no studies have been conducted to investigate the use of macroporous resin for the purification of phytochemicals from wild rice.

In order to exploit the whole grain *Z. latifolia* resources and obtain further insights into the potential health-promoting antioxidants from wild rice, an activity-guided study was carried out to evaluate the in vitro antioxidant activity of wild rice *Z. latifolia*, partially purify and separate the antioxidant constituents using a macroporous resin column, and identify and quantify individual compounds by the high-performance liquid chromatography-linear ion trap quadrupole-Orbitrap-mass spectrometry (HPLC-LTQ-Orbitrap-MS*^n^*) and ultra-performance liquid chromatography-triple quadrupole-tandem mass spectrometry (UPLC-QqQ-MS/MS).

## 2. Results and Discussion

### 2.1. Selection of Extraction Solvent

Considering the significant effect of the extraction solvent on the antioxidants extracted from plants [19,20,21], twelve different types of solvents were used to select the suitable solvent to get the maximum extraction of antioxidants from wild rice, since they were the most common ones for the extraction of antioxidants from plants [7,13,19,22]. The antioxidant activities, total flavonoid content (TFC), and total phenolic content (TPC) of each solvent extract were determined (Figure S1 in the Supplementary Materials). The results displayed that the extracts derived from ethanol, methanol, and acetone showed equivalent antioxidant activities, TFC, and TPC, which were higher than those of the other solvent extracts. Therefore, the biocompatible ethanol, with a lower cost and being less polluting, was selected as the optimal extraction solvent [22].

### 2.2. Antioxidant Activities, TFC and TPC of Ethanol Crude Extracts

Owing to the fact that pigmented rice has higher amounts of antioxidants than those of non-pigmented rice [23], and red rice *Oryza sativa* is recognized as a functional ingredient for nutraceuticals and functional foods [24], both the red and white rice *O. sativa* were used as control samples in this study. According to the results shown in Table 1, wild rice collected from Jingzhou showed the highest DPPH radical scavenging activity (45.4 ± 0.2 μmol AAE/g), followed by wild rice collected from Huai’an (20. 8 ± 0.1 μmol AAE/g). The relative low DPPH radical scavenging activities of the red and white rice *O. sativa* were observed (10.0 ± 0.0 and 1.4 ± 0.0 μmol AAE/g, respectively) (*p* < 0.05). The ABTS radical scavenging activities of Jingzhou and Huai’an wild rice (24.9 ± 0.1 and 17.0 ± 0.1 μmol AAE/g, respectively) were significantly higher than those of the control samples (red rice, 9.9 ± 0.1 μmol AAE/g; white rice, 1.8 ± 0.0 μmol AAE/g) (*p* < 0.05). In the reducing power assay, Jingzhou and Huai’an wild rice exhibited reducing powers of 63.7 ± 0.3 and 40.3 ± 0.2 μmol AAE/g, respectively, which were obviously higher than those of the control samples (21.5 ± 0.1 μmol AAE/g for red rice and 3.5 ± 0.0 μmol AAE/g for white rice) (*p* < 0.05).

The TFC and TPC of the ethanol crude extracts of wild rice and the control samples were also determined, since most antioxidant activities of plant sources correlate with the phenolic contents [21,25]. The results showed that the TFC (16.6 ± 0.2 mg QE/g) and TPC (4.8 ± 0.2 mg GAE/g) of Jingzhou wild rice were higher than those of Huai’an wild rice (12.6 ± 0.1 mg QE/g and 2.1 ± 0.0 mg GAE/g, respectively). The TFC and TPC of red rice control were 6.5 ± 0.1 mg QE/g and 1.4 ± 0.0 mg GAE/g, respectively. The white rice control contained the lowest levels of TFC (3.2 ± 0.0 mg QE/g) and TPC (1.3 ± 0.0 mg GAE/g) (Table 1) (*p* < 0.05).

These results verified that the antioxidant activity, TFC, and TPC were much higher for wild rice than for red and white rice *O. sativa*, indicating more abundant antioxidants in the former than in the latter. Furthermore, it was observed that the antioxidant profile of Jingzhou wild rice was better than that of Huai’an wild rice. This is probably attributable to the different ecological environments of the two samples, which belong to the Yangtze and Huai River basins, respectively. The level of antioxidants in the plants was influenced by various factors, such as climate, growing conditions, and ripening process. Moreover, stress conditions, such as infection by parasites and pathogens, and air pollution, may have accelerated the increase in some antioxidant metabolites [8].

### 2.3. Purification and Separation of Antioxidants

#### 2.3.1. Screening of Macroporous Resins

**Figure 1:**
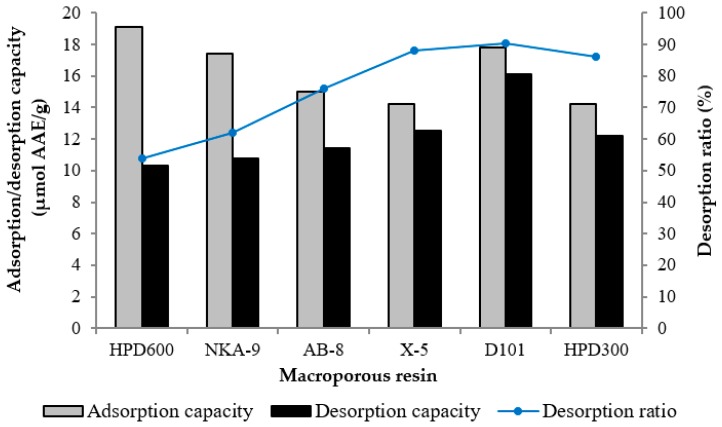
Adsorption, desorption capacities and desorption ratios of the antioxidants on different resins.

Six different resins were used in the study, to compare their adsorption and desorption performances for antioxidants from wild rice. Figure 1 shows that although the adsorption capacity of D101 resin (17.8 μmol AAE/g) was slightly lower than that of HPD600 resin (19.1 μmol AAE/g), which was the highest among the tested resins, the desorption ratio of D101 resin (90.4%) was higher than that of the other resins. Therefore, D101 resin was selected for further purification and separation. D101 resin exhibited high adsorption and desorption capacities not only because of its appropriate polarity, but also because of its large surface area and ideal average pore diameter, which correlate with the chemical feature of the adsorbate molecules [18]. If the pore diameter is too small, it can restrict the diffusion of adsorbate molecules. On the other hand, if the pore diameter is too large, the adsorbed molecules will be prone to simultaneous desorption [26]. In addition, the low desorption ratios of the polar resins HPD600 and NAK-9 indicated that some antioxidants were irreversibly adsorbed on the resins, which might be due to a strong interaction between the polar hydroxyl groups of the antioxidants and the resins [27].

#### 2.3.2. Determination of Dynamic Breakthrough Curve

To avoid losses of target compounds during the loading process on the resin column and to make the purification efficient, a dynamic breakthrough curve of the antioxidants on D101 resin was constructed. The antioxidants were almost undetectable in the effluent before 32 mL; then, the antioxidants content in the effluent increased rapidly until it reached a steady plateau at 110 mL (Figure S2 in the Supplementary Materials). According to the standard that a 10% ratio of the exit to the inlet solute concentration is defined as the breakthrough point [18], 44 mL of crude extract solution was determined as the saturated adsorption volume for the D101 resin column.

#### 2.3.3. Antioxidant Activities of Fractions 1–4

**Figure 2:**
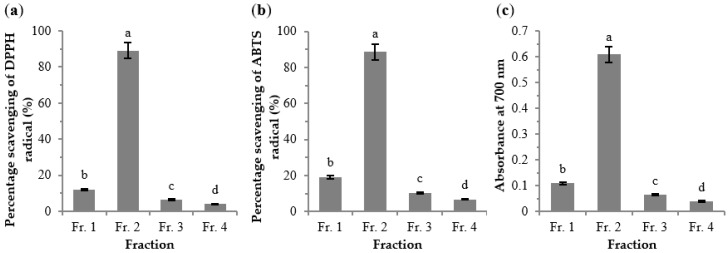
Antioxidant activities of the four fractions (*c* = 0.5 mg/mL) eluted from D101 resin column by DPPH radical (**a**), ABTS radical (**b**), and reducing power assay (**c**). The results are expressed as mean ± standard deviation (*n* = 3). Different letters above each bar within the same figure indicate significant differences (*p* < 0.05).

The crude extract of Jingzhou wild rice was subjected to a D101 resin column to obtain four fractions (Frs. 1–4). The antioxidant activities of Frs. 1–4 (at a concentration of 0.5 mg/mL) were determined (Figure 2). The percentage scavenging of DPPH and ABTS radicals and the reducing power were highest for Fr. 2, followed by Fr. 1. Frs. 3 and 4 displayed low antioxidant activities. To identify the specific compounds in the bioactive constituents of wild rice, the active Frs. 1 and 2 were analyzed by the HPLC-LTQ-Orbitrap-MS*^n^*.

### 2.4. Identification of Phenolic Acids and Their Derivatives in Fr. 1

Natural phenolic acids are distinguished by hydroxybenzoic acids and hydroxycinnamic acids structures [7]. In this study, eight hydroxybenzoic acids and their derivatives (**A1**–**7** and **A12**) and four hydroxycinnamic acids (**A8**–**11**) (the structures are shown in Figure S3 in the Supplementary Materials) were identified from Fr. 1. Table 2 presents the retention times, molecular formulas, measured and calculated deprotonated molecular ions (*m*/*z*), mass errors, and major fragment ions (*m*/*z*) for the twelve peaks in the base peak chromatogram of Fr. 1 (Figure S4A in the Supplementary Materials). According to the molecular formulas indicated by accurate molecular masses and major fragment ions from losses of molecules of CO_2_ (44 Da) and CO (28 Da) in the MS spectra, peaks **A1**–**12** were identified as gallic acid, protocatechuic acid, *p*-hydroxybenzoic acid, vanillic acid, *p*-hydroxybenzaldehyde, syringic acid, vanillin, *p*-coumaric acid, *o*-coumaric acid, ferulic acid, sinapic acid, and protocatechuic acid ethyl ester, respectively, which were confirmed by their standards.

### 2.5. Identification of Flavonoids and Phenolic Acids in Fr. 2

As one of the most important antioxidants in plants, flavonoids can be classified into different subclasses according to the substitution patterns and degrees of oxidation.

In this study, 22 compounds belonging to various metabolite families that include procyanidins (**B1**–**8**, **B11**, **B13**, **B18**, and **B19**), flavonoid glycosides (**B9**, **B10**, **B12**, **B15**–**17**), hydroxycinnamic acid derivatives (**B14** and **B21**), flavonols (**B20**), and flavones (**B22**) were identified from Fr. 2 (Table 3). The structures of the compounds and the base peak chromatogram of Fr. 2 are provided in Figures S3 and S4B in the Supplementary Materials, respectively.

#### 2.5.1. Procyanidins

Procyanidins, the oligomers and polymers of catechin and epicatechin, can be divided into two different structure types. In the more common B-type procyanidins, the (epi)catechin units are connected through a single bond between C-4 of the upper unit and C-6 or C-8 of the lower unit. The A-type procyanidins differ from the B-type by having an additional bond between adjacent (epi)catechin units that connects C-2 of the upper unit via an oxygen atom to C-7 or the less abundant C-5 of the lower unit [28].

Red rice *O. sativa* has been demonstrated to be a natural source of procyanidins [25]. However, procyanidins from wild rice have not been well studied with the exception of one primary research on North American wild rice, in which only catechin, epicatechin, and four procyanidin oligomers were identified [13].

**Figure 3:**
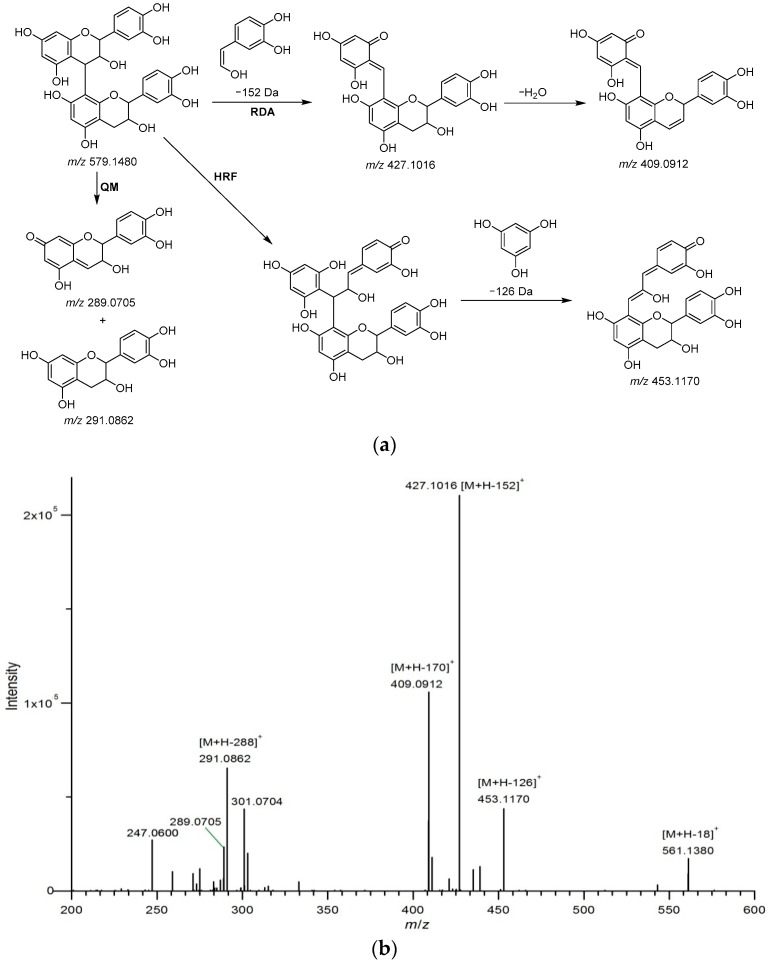
Fragmentation pathway (**a**) and MS^2^ spectrum (**b**) of B-type procyanidin dimers. The fragment mechanisms are RDA (retro-Diels-Alder), HRF (heterocyclic ring fission), and QM (quinone methide) cleavage.

Twelve procyanidins were identified from Fr. 2. Peaks **B1**–**3** showed a protonated molecular ion at *m*/*z* 579.1480 [M + H]^+^ with fragment ions at *m*/*z* 291.0862 [M + H − 288]^+^ generated by the loss of an (epi)catechin unit through quinone methide (QM) cleavage of the interflavan bond, and 561.1380 [M + H − 18]^+^ from the loss a water molecular [28], and three noticeable fragment ions of B-type procyanidin dimers formed by losses of 126, 152, and 170 Da [29]. As is shown in Figure 3, the fragment at *m*/*z* 453.1170 [M + H − 126]^+^ corresponded to the elimination of a phloroglucinol molecule through heterocyclic ring fission (HRF). The fragments at *m*/*z* 427.1016 [M + H − 152]^+^ and 409.0912 [M + H − 170]^+^ originated from a retro-Diels–Alder (RDA) reaction, and the latter eliminated a water molecule. The fragment at *m*/*z* 289.0705 [M + H − 290]^+^ was generated by QM cleavage of the interflavan bond [28,30]. Accordingly, peaks **B1**–**3** were respectively identified as procyanidins B1, B2, and B3, based on the standards. Peaks **B4** (*m*/*z* 307.0818 [M + H]^+^), **B5** and **B6** (*m*/*z* 291.0862 [M + H]^+^) were respectively identified as epigallocatechin, catechin, and epicatechin, using the standards. Peak **B7** (*m*/*z* 1153.2559 [M + H]^+^) exhibited fragments at *m*/*z* 865.1963 [M + H − 288]^+^, 713.1592 [M + H − 288 − 152]^+^, and 577.1334 [M + H – 288 − 288]^+^ (from QM cleavage and RDA reaction), being identified as an A-type procyanidin tetramer [30]. The protonated molecular ion at *m*/*z* 577.1326 [M + H]^+^ and fragments at *m*/*z* 559.1220 [M + H − 18]^+^ (loss of a water molecule), 451.1013 [M + H − 126]^+^ (loss of a phloroglucinol molecule), and 425.0858 [M + H − 152]^+^ (from RDA reaction) led to the assignment of peak **B8** as an A-type procyanidin dimer [29]. Furthermore, a B-type procyanidin tetramer (peak **B11**, *m*/*z* 1155.2715 [M + H]^+^) showing fragments at *m*/*z* 1029.2438 [M + H − 126]^+^ (loss of a phloroglucinol molecule), 1003.2283 [M + H − 152]^+^ (from RDA reaction), and 867.2122 [M + H − 288]^+^ (loss of an (epi)catechin unit) was identified [31]. In addition, peaks **B13** (*m*/*z* 865.1955 [M + H]^+^) and **B18** (*m*/*z* 863.1792 [M + H]^+^), with the typical fragment ions of procyanidin oligomers (Table 3), were assigned as A-type procyanidin trimers, on the basis of literature data [30,32]. Peak **B19** (*m*/*z* 867.2138 [M + H]^+^) was identified as procyanidin C1 using the standard.

#### 2.5.2. Flavonoid Glycosides

**Figure 4:**
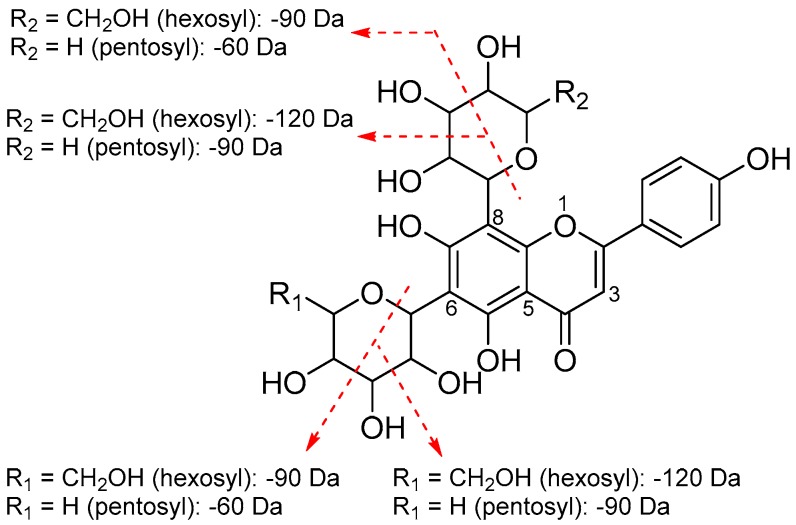
Fragmentation pattern of 6,8-di-*C*-diglycosylated apigenins.

Natural flavonoids are usually found in *O*-glycoside and *C*-glycoside forms. Six flavonoid glycosides were identified from Fr. 2. Peak **B9** (*m*/*z* 611.1597 [M + H]^+^) with a major fragment at *m*/*z* 303.0485 [M + H − 308]^+^ (loss of a rutinose moiety) was identified as rutin, based on the standard. Peak **B10** had a protonated molecular ion at *m*/*z* 451.1222 [M + H]^+^, dissociating to yield fragments at *m*/*z* 289.0714 [M + H − 162]^+^ (loss of a hexose moiety), 271.0608 [M + H − 162 − 18]^+^ and 245.0818 [M + H − 162 − 44]^+^ (for the presence of eriodictyol), and was identified as eriodictyol 7-*O*-hexoside [33]. Generally, in the MS spectrum, the characteristic losses for *O*-glycosides are 162 (hexose), 146 (deoxyhexose), and 132 Da (pentose), which correspond to the complete losses of the sugar moieties produced by cleavage at *O*-glycosidic bonds. In contrast to *O*-glycosides, the losses of 120, 90, and 60 Da, formed by cross-cleavages within sugar moieties, and an additional 18 Da representing a water molecule loss, could be diagnostic for *C*-glycosides. In most cases, *C*-glycosylation was found at C-6 and C-8 positions of the flavonoid aglycone [13]. Figure 4 illustrates the fragmentation pattern of the *C*-glycosylated flavonoids (peaks **B12**, **B15**–**17**) identified from Fr. 2. Peak **B12** (*m*/*z* 595.1647 [M + H]^+^) produced fragments from losses of water molecules (*m*/*z* 577.1543 [M + H − 18]^+^ and 559.1436 [M + H − 36]^+^), and 475.1226 [M + H − 120]^+^ (formed by cross-ring cleavage of the hexose moiety), indicating the presence of a *C*-diglycosylated flavonoid [34]. The aglycone of apigenin was deduced from the occurrence of a fragment at *m*/*z* 355.0808 [M + H − 120 − 120]^+^ [13]. Therefore peak **B12** was identified as 6,8-di-*C*-hexosyl apigenin. Peaks **B15** (*m*/*z* 565.1534 [M + H]^+^) and **B16** (*m*/*z* 565.1544 [M + H]^+^) produced fragments at *m*/*z* 475.1123 [M + H − 90]^+^, 445.1124 [M + H − 120]^+^, 415.1020 [M + H − 60 − 90]^+^, 547.1437 [M + H − 18]^+^, and 529.1331 [M + H − 36]^+^, which suggested that the two compounds were *C*-glycoside comprising one hexosyl and one pentosyl moiety. Furthermore, a fragment of peaks **B15** and **B16** at *m*/*z* 355.0808 [M + H − 120 − 90]^+^ representing apigenin was also observed. Accordingly, the two compounds were assigned as hexosyl-pentosyl apigenin. According to earlier reports [13,34], in the MS^2^ spectrum of 6-*C*-hexosyl-8-*C*-pentosyl apigenin, the ion [M + H − 120]^+^ formed by cross-ring cleavage of the hexose moiety, has a higher relative intensity than that of the ion [M + H − 90]^+^ from the cross-ring cleavages of both the hexose and pentose moieties, and an opposite result should be obtained for 6-*C*-pentosyl-8-*C*-hexosyl apigenin; hence, peaks **B15** and **B16** were concluded to be 6-*C*-hexosyl-8-*C*-pentosyl apigenin and 6-*C*-pentosyl-8-*C*-hexosyl apigenin, respectively, because higher relative intensities of 445.1124 [M + H − 120]^+^ for peak **15** and 475.1123 [M + H − 90]^+^ for peak **16** were observed. Peak **B17** (*m*/*z* 535.1431 [M + H]^+^) exhibited fragments from losses of water molecules (*m*/*z* 517.1345 [M + H − 18]^+^ and 499.1221 [M + H − 36]^+^), from cross-ring cleavage of the pentose moiety (*m*/*z* 475.1225 [M + H − 60]^+^ and 445.1123 [M + H − 90]^+^), and for the presence of apigenin (*m*/*z* 355.0810 [M + H − 90 − 90]^+^), and was hence identified as 6,8-di-*C*-pentosyl apigenin [34].

#### 2.5.3. Others

Peak **B14** (*m*/*z* 373.1134 [M + H]^+^) was identified as hydroferulic acid 4-*O*-glucuronide, on the basis of the fragments at *m*/*z* 355.1022 [M + H − 18]^+^ (loss of a water molecule) and 197.0807 [M + H − 176]^+^ (loss of a glucuronic acid moiety) [35]. Peak **B20** (*m*/*z* 303.0494 [M + H]^+^) was identified as quercetin, using the standard. 3,4,5-Trimethoxycinnamic acid (Peak **B21**, *m*/*z* 239.0915 [M + H]^+^), exhibiting fragments at *m*/*z* 224.0684 [M + H − 15]^+^ (loss of a methyl) and 195.1019 [M + H − 44]^+^ (loss of a CO_2_ molecule), was identified based on the literature [36]. Peak **B22** (*m*/*z* 331.0796 [M + H]^+^), with fragments at *m*/*z* 316.0568 [M + H − 15]^+^ (loss of a methyl) and 301.0340 [M + H − 30]^+^ (loss of two methyls), was tentatively assigned as tricin [37].

A total of 34 phenolic compounds were identified from Fr. 1 (mainly low molecular weight phenolic acids) and Fr. 2 (mainly flavonoids). Strong and positive correlations between the phenolics content and antioxidant activities of wild rice measured by DPPH and ABTS radical methods, have been revealed in an earlier report [8]. To the best of our knowledge, 14 compounds, including nine procyanidins (**B1**–**3**, **B7**, **B8**, **B11**, **B13**, **B18**, and **B19**), two flavonoid glycosides (**B10** and **B16**), two hydroxycinnamic acid derivatives (**B14** and **B21**), and one flavone (**B22**), have been identified from wild rice for the first time, in this study. All the phenolic acids and flavonoids from wild rice, reported previously, are summarized in Table S1 in the Supplementary Materials.

### 2.6. Quantification of Antioxidants

**Figure 5:**
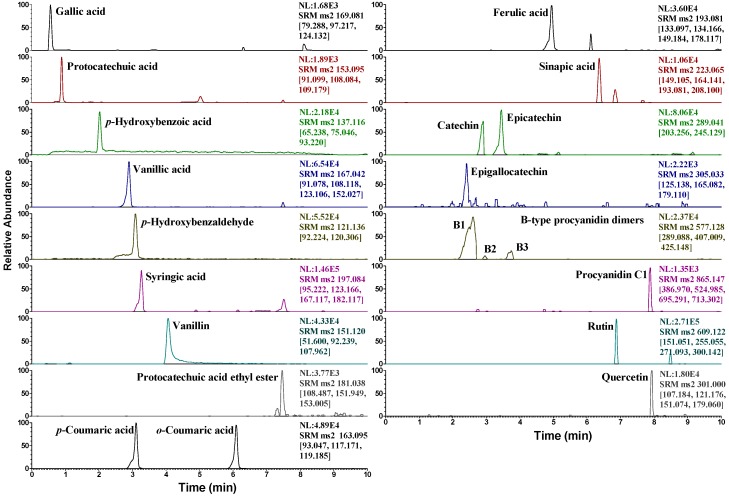
MRM chromatograms of Jingzhou wild rice containing 21 target compounds.

The contents of the compounds in two wild rice and control samples were determined using the multiple reaction monitoring (MRM) mode of UPLC-QqQ-MS/MS. Due to the lack of available standards, only 21 compounds were quantified. The representative MRM chromatograms of Jingzhou wild rice are shown in Figure 5. The results (Table 4) revealed that significantly higher concentrations of phenolic acids and flavonoids were detected in wild rice than in the control samples. Ferulic acid, followed by gallic acid and sinapic acid, was the most abundant phenolic in both the wild rice samples. Huai’an wild rice had higher amounts of ferulic acid (189.7 ± 1.0 μg/g) and gallic acid (167.1 ± 0.6 μg/g) than those of Jingzhou wild rice (121.1 ± 0.8 and 64.6 ± 0.4 μg/g, respectively), whereas a smaller amount of sinapic acid was detected in Huai’an wild rice (26.8 ± 0.3 μg/g) than in Jingzhou wild rice (59.4 ± 0.4 μg/g). As for the content of total phenolic acids, a higher value of 472.5 μg/g was assessed in Huai’an wild rice than in Jingzhou wild rice (349.3 μg/g). A high content of procyanidins was detected in both the wild rice samples. The results established the following order of procyanidin content in wild rice: epicatechin > procyanidin C1 > catechin > procyanidin B1 > epigallocatechin > procyanidin B3 > procyanidin B2. It was worth noting that, in contrast to total phenolic acids content, the total procyanidins content was higher in Jingzhou wild rice (126.2 μg/g) than in Huai’an wild rice (86.4 μg/g). The control sample red rice contained a total procyanidins content of 28.9 μg/g. No procyanidins were detected in the white rice control. The aforementioned information together with the order of the antioxidant activities of the three samples (Jingzhou wild rice > Huai’an wild rice > red rice > white rice) implied that the antioxidant activity of wild rice may mainly be associated with the accumulation of flavonoids, especially procyanidins, which were found in Fr. 2, the most active fraction. Supporting the aforementioned speculation, an earlier study reported that phenolic acids only constitute a small portion of antioxidant compounds in wild rice, and flavonoids and other phytochemicals may contribute to the bulk of its antioxidant capacity [7].

## 3. Materials and Methods

### 3.1. Plant Materials and Chemicals

Whole grains of wild rice (*Z. latifolia*) were hand-harvested from Jingzhou (Hubei Province, China) and Huai’an (Jiangsu Province, China) in September 2017. The whole grains of red and white rice *O. sativa* collected from Huai’an was used as control samples. All the freeze-dried rice grains were ground to a fine powder in a mechanical grinder and sieved through a 0.45 mm sifter.

Folin-Ciocalteu reagent, DPPH (2,2-diphenyl-1-picrylhydrazyl) (97% purity), ABTS (2,2′-azino-bis(3-ethylbenothiazoline-6-sulfonic acid) diammonium salt (98% purity), and all the phenolic acid and flavonoid standards (≥99% purity) were purchased from Sigma-Aldrich Chemical Co. (St. Louis, MO, USA). Precoated silica gel plates GF254 purchased from Qingdao Haiyang Chemical Co. Ltd. (Qingdao, China) were used for thin layer chromatography analyses. The LC-MS grade solvents (99.9% purity) were purchased from Sigma-Aldrich Chemical Co (St. Louis, MO, USA). Six macroporous resins, including HPD600, NKA-9, AB-8, X-5, D101, and HPD300, with different physical properties (Table S2 in the Supplementary Materials), were purchased from Solarbio Science & Technology Co. Ltd. (Beijing, China). Before the experiments, the resins were pretreated as previously reported [38].

### 3.2. Extraction

Twelve different solvents, including six native solvents (ethanol, methanol, acetone, 70% aqueous ethanol, 70% aqueous methanol, and 70% aqueous acetone) alone and acidified with 1% (*v*/*v*) acetic acid, were used to obtain antioxidants from wild rice. The rice flour was extracted twice with the solvent in an ultrasonic cleaner for 1 h, at 40 °C and a ratio of liquid to solid of 50 mL/g [7,13]. The mixture was centrifuged at 5000 rpm for 20 min. The supernatants were combined and used as the crude extract to determine the antioxidant activities, TFC, and TPC. The crude extract derived from ethanol, which was concentrated in vacuum, was stored at −20 °C for further purification and identification.

### 3.3. Evaluation of Antioxidant Activities

The in vitro antioxidant activities were evaluated by DPPH [39] and ABTS radical [40], and reducing power [39] methods, with ascorbic acid as the reference. The results were expressed as micromoles of ascorbic acid equivalents (AAE) per g of rice on a dry weight basis (μmol AAE/g). The antioxidants content measured by the DPPH radical method was expressed as micromoles of ascorbic acid equivalents per mL of the sample solution (μmol AAE/mL).

### 3.4. Determination of TFC and TPC

The TFC and TPC were measured according to previously described methods [41]. TFC was calculated using a standard quercetin curve and expressed as mg of quercetin equivalents (QE) per g of rice (mg QE/g). TPC was expressed as mg of gallic acid equivalents (GAE) per g of rice (mg GAE/g).

### 3.5. Screening of Macroporous Resins

Macroporous resins were screened by static adsorption and desorption tests, according to a previously described method [17], with minor modifications. The ethanol crude extract of wild rice was dissolved in distilled water to give a crude extract solution (antioxidants content, 0.50 μmol AAE/mL). 1.0 g of the pretreated resin was put into a 200 mL flask and then 50 mL of the crude extract solution was added. After shaken on an immersion oscillator (120 rpm), at room temperature for 24 h to reach adsorption equilibrium, the resins were washed with deionized water and then desorbed with 50 mL of 70% (*v*/*v*) aqueous ethanol in the flask, which was continually shaken (120 rpm) at room temperature for 24 h. The screening of resins was based on the capacities of adsorption and desorption, and the desorption ratio, which were quantified according to Equations (1)–(3):

*Q*_a_ = (*C*_0_ − *C*_a_) × *V*_0_/*m* (1)

*Q*_d_ = *C*_d_ × *V*_d_/*m* (2)

*D* = *C*_d_ × *V*_d_/[(*C*_0_ − *C*_a_) × *V*_0_] × 100 (3)

where *Q*_a_ is the adsorption capacity at adsorption equilibrium (μmol AAE/g dry resin); *Q*_d_ is the desorption capacity after adsorption equilibrium (μmol AAE/g dry resin); *C*_0_, *C*_a_, and *C*_d_ represent the antioxidants contents of the solution at initial, absorption equilibrium, and desorption status, respectively (μmol AAE/mL); *V*_0_ and *V*_d_ are the volumes of the initial sample and desorption solution (mL), respectively; *m* is the dry weight of resin (g); and *D* means the desorption ratio (%).

### 3.6. Determination of Dynamic Breakthrough Curve

The dynamic breakthrough curve of antioxidants on the D101 resin column was constructed using a dynamic adsorption test, which was performed on a glass column (16 × 300 mm) wet-packed with 15.0 g of D101 resin. The bed volume (BV) of the resin was 20 mL. The adsorption process was carried out by overloading the column with the crude extract solution (antioxidants content, 0.50 μmol AAE/mL) at a flow rate of 2 BV/h. The effluent liquids were collected by an automatic fraction collector (4 mL for each tube) and the antioxidants content for each tube was analyzed.

### 3.7. D101 Macroporous Resin Column Chromatography

The crude extract solution (44 mL) was subjected to a glass column (16 × 300 mm) wet-packed with 15.0 g of D101 resin. After reaching adsorption equilibration, the resins adsorbed with the sample were initially washed with deionized water (2 BV), and then eluted successively with 10%, 20%, 30%, 40%, 50%, 60%, 70%, 80%, 90%, and 100% (*v*/*v*) aqueous ethanol (5 BV for each, 2 BV/h). In sequence, the effluents of the different eluents were collected and then concentrated and pooled to obtain four fractions (Frs. 1–4) based on their TLC and HPLC fingerprint chromatograms (Figure S5 in the Supplementary Materials). In general, the 10% ethanol eluent was collected as Fr. 1. The 20–30% ethanol eluents were combined as Fr. 2. The eluents of 40–60% ethanol gave Fr. 3. Lastly, the eluents of 70–100% ethanol produced Fr. 4.

### 3.8. HPLC-LTQ-Orbitrap-MS^n^ Analysis

An Accella HPLC instrument with a diode-array detector and an autosampler, coupled with an linear ion trap quadrupole Orbitrap XL mass spectrometer (Thermo Scientific, San Jose, CA, USA), was used for the compounds identification. During the analysis, 5 μL of sample was injected and eluted through an Agilent poroshell 120 EC-C18 column (2.7 µm, 4.6 × 150 mm) with a gradient mobile phase consisting of 0.1% (*v*/*v*) acetic acid in acetonitrile (solvent A) and 0.1% (*v*/*v*) acetic acid in water (solvent B) at a flow rate of 0.3 mL/min. The solvent system used for Fr. 1 was as follows: 0–15 min, 5–10% A; 15–20 min, 10–15% A; 20–25 min, 15–20% A; 25–30 min, 20–5% A. The solvent system used for Fr. 2 was as follows: 0–10 min, 5–10% A; 10–25 min, 10–15% A; 25–30 min, 15–40% A; 30–35 min, 40–5% A.

For mass detection, an electrospray ionization (ESI) source was operated in negative mode with a scan range from *m*/*z* 50 to 1000 for Fr. 1, and positive mode with a scan range from *m*/*z* 150 to 2000 for Fr. 2. The capillary temperature was 350 °C. Nitrogen was used as the sheath gas and auxiliary gas, and the gas flow was set at 30 arb and 5 arb, respectively. The spray voltage was 4000 V for the positive mode and 3000 V for the negative mode. The collision energy was 35%, to adjust collision-induced dissociation for the best performance. The Xcalibur 2.1 software (Thermo Scientific) was used for data analysis.

The identification of compounds was determined on the basis of their retention times, UV spectra, and accurate mass data; a compound was positively identified when all the data matched those of the standard. Those with no available standards were tentatively identified by comparison with literature data. The mass errors for the quasi-molecular ions of all the identified compounds were within ±5 ppm.

### 3.9. UPLC-QqQ-MS/MS Analysis

The quantitation of compounds was performed on a UPLC-QqQ-MS/MS system, which consisted of a Waters ACQUITY H-CLASS UPLC instrument equipped with an autosampler (Waters, Milford, MA, USA), and a TSQ Quantum Ultra triple quadrupole tandem mass spectrometer (Thermo Scientific). The analytes were chromatographed by injecting 2 µL of sample into a Waters ACQUITY UPLC BEH C18 column (1.7 µm, 2.1 × 50 mm). The binary mobile phase consisted of 0.1% (*v*/*v*) acetic acid in acetonitrile (solvent A) and 0.1% (*v*/*v*) acetic acid in water (solvent B), and the solvent gradient was as follows: 0–5 min, 5–10% A; 5–7 min, 10–20% A; 7–8 min, 20–60% A; 8–9 min, 60–100% A; 9–10 min, 100–5% A. The flow rate was 0.3 mL/min.

An ESI source was used with negative ions in the MRM mode. The optimized ion spray voltage was 3000 V. The vaporizer and capillary temperature were 350 and 320 °C, respectively. Nitrogen was used as the sheath gas (30 arb) and auxiliary gas (5 arb), and argon was used as the collision gas (1.5 mTorr). The collision energy was optimized individually for each transition. Data acquisition and processing were performed using the Xcalibur 3.1 software (Thermo Scientific). The ion transitions, optimized MS parameters, and linear relationships of the 21 external standards are listed in Table S3 in the Supplementary Materials.

### 3.10. Statistical Analysis

All the assays were performed at least in triplicate and data were expressed as mean ± standard error. One-way analysis of variance (ANOVA) followed by Duncan’s multiple range test were used to determine statistically different values at a significance level of *p* < 0.05. All the statistical analyses were performed using SPSS 19.0 for Windows (SPSS Inc., Chicago, IL, USA).

## 4. Conclusions

During this first investigation on the antioxidants from wild rice *Z. latifolia*, the ethanol extract of this species was demonstrated to be a potent source of natural antioxidants, that can be enriched in 10% (Fr. 1) and 20–30% ethanol-eluted fractions (Fr. 2) obtained by D101 macroporous resin column chromatography. The HPLC-LTQ-Orbitrap-MS*^n^* analysis of the active fractions led to the identification of 34 phenolic acids and flavonoids, among which 14 compounds were firstly encountered in wild rice. We found that the active Frs. 1 and 2 mainly contained phenolic acids and flavonoids (including procyanidins and flavonoid glycosides), respectively. These first respective enrichments of phenolic acids and flavonoids provide references for the application of the two families of potential natural antioxidants from wild rice. Compared with phenolic acids, flavonoids may contribute more to the antioxidant activity of wild rice. This study offers new insights into the functional components of wild rice and may advance the understanding and development of the abundant but underutilized *Z. latifolia* resources in East Asia.

## Figures and Tables

**Table 1:** Antioxidant activities, total flavonoid content (TFC), and total phenolic content (TPC) of ethanol crude extracts from wild rice and control samples.

Sample	DPPH (μmol AAE/g)	ABTS (μmol AAE/g)	Reducing Power (μmol AAE/g)	TFC (mg QE/g)	TPC (mg GAE/g)
Wild rice (Jingzhou)	45.4 ± 0.2 ^a^	24.9 ± 0.1 ^a^	63.7 ± 0.3 ^a^	16.6 ± 0.2 ^a^	4.8 ± 0.2 ^a^
Wild rice (Huai’an)	20.8 ± 0.1 ^b^	17.0 ± 0.1 ^b^	40.3 ± 0.2 ^b^	12.6 ± 0.1 ^b^	2.1 ± 0.0 ^b^
Red rice (*O. sativa*)	10.0 ± 0.0 ^c^	9.9 ± 0.1 ^c^	21.5 ± 0.1 ^c^	6.5 ± 0.1 ^c^	1.4 ± 0.0 ^c^
White rice (*O. sativa*)	1.4 ± 0.0 ^d^	1.8 ± 0.0 ^d^	3.5 ± 0.0 ^d^	3.2 ± 0.0 ^d^	1.3 ± 0.0 ^c^

Values are expressed as mean ± standard error (*n* = 3). Values with different letters in the same column indicate significant differences (*p* < 0.05). AAE, ascorbic acid equivalents; QE, quercetin equivalents; GAE, gallic acid equivalents.

**Table 2:** Identification of phenolic acids and their derivatives in Fr. 1.

Peak ^a^	Compound ^b^	*t*_R_ (min)	Formula	[M − H]^−^ (*m*/*z*)			Fragment Ion (*m*/*z*)
				Measured	Calculated	Error (ppm)	
	Hydroxybenzoic acids and their derivatives						
**A1**	Gallic acid	3.07	C_7_H_6_O_5_	169.0141	169.0142	−0.86	125.0244
**A2**	Protocatechuic acid	5.70	C_7_H_6_O_4_	153.0190	153.0193	−2.15	109.0129
**A3**	*p*-Hydroxybenzoic acid	9.08	C_7_H_6_O_3_	137.0243	137.0244	−0.59	93.0340, 65.0394
**A4**	Vanillic acid	11.82	C_8_H_8_O_4_	167.0346	167.0350	−2.31	123.0450
**A5**	*p*-Hydroxybenzaldehyde	12.00	C_7_H_6_O_2_	121.0291	121.0295	−3.51	-
**A6**	Syringic acid	13.76	C_9_H_10_O_5_	197.0447	197.0455	−3.94	153.0551, 123.0449
**A7**	Vanillin	15.19	C_8_H_8_O_3_	151.0398	151.0401	−1.83	136.0163, 107.0500
**A12**	Protocatechuic acid ethyl ester	26.25	C_9_H_10_O_4_	181.0504	181.0506	−0.98	153.0553
	Hydroxycinnamic acids						
**A8**	*p*-Coumaric acid	19.93	C_9_H_8_O_3_	163.0397	163.0401	−2.39	119.0500
**A9**	*o*-Coumaric acid	20.82	C_9_H_8_O_3_	163.0396	163.0401	−2.87	119.0500
**A10**	Ferulic acid	23.91	C_10_H_10_O_4_	193.0503	193.0506	−2.05	149.0602
**A11**	Sinapic acid	25.11	C_11_H_12_O_5_	223.0603	223.0612	−4.08	179.0709, 164.0471

^a^ Peaks were numbered according to their order of elution from the lowest to the highest retention times. ^b^ Identification of the compounds was confirmed by authentic standards. *t*_R_, retention time.

**Table 3:** Identification of flavonoids and phenolic acids in Fr. 2.

Peak ^a^	Compound	*t*_R_ (min)	Formula	[M + H]^+^ (*m*/*z*)			Fragment Ion (*m*/*z*)
				Measured	Calculated	Error (ppm)	
	Procyanidins						
**B1**	Procyanidin B1 ^b^	7.63	C_30_H_26_O_12_	579.1480	579.1497	−2.87	561.1380, 453.1170, 427.1016, 409.0912, 291.0862, 289.0705
**B2**	Procyanidin B2 ^b^	8.34	C_30_H_26_O_12_	579.1480	579.1497	−2.87	561.1380, 453.1170, 427.1016, 409.0912, 291.0862, 289.0705
**B3**	Procyanidin B3 ^b^	9.55	C_30_H_26_O_12_	579.1481	579.1497	−2.69	561.1380, 453.1170, 427.1016, 409.0912, 291.0862, 289.0705
**B4**	Epigallocatechin ^b^	10.10	C_15_H_14_O_7_	307.0818	307.0812	1.89	181.0490
**B5**	Catechin ^b^	11.01	C_15_H_14_O_6_	291.0862	291.0863	−0.56	273.0747, 165.0544, 139.0836
**B6**	Epicatechin ^b^	11.08	C_15_H_14_O_6_	291.0862	291.0863	−0.56	273.0347, 165.0544, 139.0836
**B7**	A-type procyanidin tetramer ^c^	12.97	C_60_H_48_O_24_	1153.2559	1153.2608	−4.30	865.1963, 713.1592, 577.1334
**B8**	A-type procyanidin dimer ^c^	15.79	C_30_H_24_O_12_	577.1326	577.1341	−2.86	559.1220, 451.1013, 425.0858
**B11**	B-type procyanidin tetramer ^c^	17.07	C_60_H_50_O_24_	1155.2715	1155.2765	−4.41	1029.2438, 1003.2283, 867.2122
**B13**	A-type procyanidin trimer ^c^	17.96	C_45_H_36_O_18_	865.1955	865.1974	−2.27	713.1488, 695.1382, 577.1543
**B18**	A-type procyanidin trimer ^c^	21.07	C_45_H_34_O_18_	863.1792	863.1818	−2.95	845.1689, 711.1322, 693.1221
**B19**	Procyanidin C1 ^b^	22.15	C_45_H_38_O_18_	867.2138	867.2131	0.92	715.1660, 697.1447
	Flavonoid glycosides						
**B9**	Rutin ^b^	16.10	C_27_H_30_O_16_	611.1597	611.1607	−1.69	303.0485
**B10**	Eriodyctyol 7-*O*-hexoside ^c^	16.81	C_21_H_22_O_11_	451.1222	451.1235	−2.91	289.0714, 271.0608, 245.0818
**B12**	6,8-di-*C*-hexosyl apigenin ^c^	17.36	C_27_H_30_O_15_	595.1647	595.1657	−1.80	577.1543, 559.1436, 475.1226, 355.0808
**B15**	6-*C*-hexosyl-8-*C*-pentosyl apigenin ^c^	18.79	C_26_H_28_O_14_	565.1534	565.1552	−3.13	547.1437, 529.1331, 475.1123, 445.1124, 415.1020, 355.0808
**B16**	6-*C*-pentosyl-8-*C*-hexosyl apigenin ^c^	19.35	C_26_H_28_O_14_	565.1544	565.1552	−1.39	547.1437, 529.1331, 475.1123, 445.1124, 415.1020, 355.0808
**B17**	6,8-di-*C*-pentosyl apigenin ^c^	20.69	C_25_H_26_O_13_	535.1431	535.1446	−3.86	517.1345, 499.1221, 475.1225, 445.1123, 355.0810
	Others						
**B14**	Dihydroferulic acid 4-*O*-glucuronide ^c^	18.28	C_16_H_20_O_10_	373.1134	373.1129	1.33	355.1022, 197.0807
**B20**	Quercetin ^b^	24.09	C_15_H_10_O_7_	303.0494	303.0499	−1.61	181.0128, 153.0178
**B21**	3,4,5-Trimethoxycinnamic acid ^c^	26.13	C_12_H_14_O_5_	239.0915	239.0914	0.26	224.0684, 195.1019
**B22**	Tricin ^c^	28.05	C_17_H_14_O_7_	331.0796	331.0812	−4.90	316.0568, 301.0340

^a^ Peaks were numbered according to their order of elution from the lowest to the highest retention times. ^b^ Identification of the compound was confirmed by authentic standard. *^c^* Compound was tentatively identified by comparison with literature data. *t*_R_, retention time.

**Table 4:** Quantification results (μg/g rice) of phenolic compounds in wild rice and control samples.

Compound	Wild Rice (Jingzhou)	Wild Rice (Huai’an)	Rice (*O. sativa*)	
			Red	White
Phenolic acids				
Gallic acid	64.6 ± 0.4 ^b^	167.1 ± 0.6 ^a^	1.1 ± 0.0 ^c^	0.2 ± 0.0 ^d^
Protocatechuic acid	15.6 ± 0.2 ^a^	12.9 ± 0.1 ^b^	7.8 ± 0.1 ^c^	nd
*p*-Hydroxybenzoic acid	11.1 ± 0.1 ^a^	7.1 ± 0.1 ^b^	nd	0.8 ± 0.0 ^c^
Vanillic acid	17.8 ± 0.2 ^a^	6.3 ± 0.1 ^b^	nd	1.3 ± 0.0 ^c^
*p*-Hydroxybenzaldehyde	15.6 ± 0.1 ^a^	12.1 ± 0.1 ^b^	nd	nd
Syringic acid	19.5 ± 0.2 ^a^	5.1 ± 0.0 ^b^	nd	0.9 ± 0.0 ^c^
Vanillin	13.0 ± 0.1 ^b^	22.3 ± 0.2 ^a^	1.0 ± 0.0 ^c^	nd
Protocatechuic acid ethyl ester	2.0 ± 0.0 ^b^	6.1 ± 0.0 ^a^	nd	nd
*p*-Coumaric acid	6.7 ± 0.0 ^a^	7.0 ± 0.1 ^a^	1.1 ± 0.0 ^b^	1.2 ± 0.0 ^b^
*o*-Coumaric acid	2.9 ± 0.0 ^b^	10.0 ± 0.1 ^a^	nd	nd
Ferulic acid	121.1 ± 0.8 ^b^	189.7 ± 1.0 ^a^	12.4 ± 0.3 ^c^	10.9 ± 0.1 ^d^
Sinapic acid	59.4 ± 0.4 ^a^	26.8 ± 0.3 ^b^	3.2 ± 0.0 ^d^	4.6 ± 0.0 ^c^
**Total phenolic acids**	**349.3**	**472.5**	**26.6**	**19.9**
Flavonoids				
Catechin	21.3 ± 0.3 ^a^	15.6 ± 0.2 ^b^	6.6 ± 0.1 ^c^	nd
Epicatechin	43.3 ± 0.5 ^a^	24.3 ± 0.3 ^b^	3.5 ± 0.0 ^c^	nd
Epigallocatechin	10.0 ± 0.2 ^a^	7.5 ± 0.2 ^b^	nd	nd
Procyanidin B1	13.0 ± 0.2 ^a^	10.2 ± 0.1 ^b^	7.0 ± 0.1 ^c^	nd
Procyanidin B2	5.0 ± 0.1 ^a^	5.5 ± 0.1 ^a^	2.4 ± 0.0 ^c^	nd
Procyanidin B3	9.4 ± 0.1 ^a^	6.3 ± 0.1 ^b^	3.4 ± 0.0 ^c^	nd
Procyanidin C1	24.2 ± 0.1 ^a^	17.0 ± 0.2 ^b^	6.0 ± 0.1 ^c^	nd
**Total procyanidins**	**126.2**	**86.4**	**28.9**	-
Rutin	103.7 ± 0.7 ^a^	83.6 ± 0.5 ^b^	20.8 ± 0.2 ^c^	15.7 ± 0.2 ^d^
Quercetin	15.4 ± 0.1 ^b^	44.1 ± 0.2 ^a^	16.6 ± 0.2 ^c^	nd

Values are mean ± standard error (*n* = 5). Values with different letters in the same row indicate significant differences (*p* < 0.05). nd, not detected.

## Supplementary Materials

Supplementary materials are available online. Table S1: Phenolic acids and flavonoids found in wild rice in previous reports; Table S2: Physical properties of six macroporous resins; Table S3: The ion transitions, optimized MS parameters, and linear relationships of the standards in UPLC-QqQ-MS/MS analysis; Figure S1: The radical scavenging activities of DPPH (A) and ABTS (B), reducing power (C), total flavonoid content (TFC) (D), and total phenolic content (TPC) (E) of different solvent extracts of wild rice collected from Jingzhou; Figure S2: Dynamic breakthrough curve of antioxidants on D101 resin column; Figure S3: Structures of phenolic compounds identified in wild rice; Figure S4: Base peak chromatograms of Frs. 1 (A) and 2 (B) in HPLC-LTQ-Orbitrap-MS*^n^* analysis; Figure S5: HPLC fingerprint chromatograms at 360 nm (A) and 280 nm (B) of the four fractions (Frs. 1–4) eluted from D101 resin column. The gradient solvent system consisting of A (methanol) and B (water containing 0.1% acetic acid, *v*/*v*) was as follows: 0–10 min, 5–10% A; 10–30 min, 10–15% A; 30–40 min, 15–20% A; 40–50 min, 20–25% A; 50–60 min, 25–35% A; 60–70 min, 35–90% A; 70–80 min, 90–60% A.
