# Supplementary material for: Partial Purification, Identification, and Quantitation of Antioxidants from Wild Rice (Zizania latifolia)
Source: Molecules. 2018 Oct 26;23(11):2782. doi: 10.3390/molecules23112782 (PMC6278310; doi:10.3390/molecules23112782)
Supplement: Supplementary file 1 [file molecules-23-02782-s001.pdf]

Supplementary Materials for

Partial Purification, Identification, and Quantitation of Antioxidants from Wild Rice *Zizania latifolia*

Mei-Jun Chu, Xin-Min Liu, Ning Yan, Feng-Zhong Wang, Yong-Mei Du * and Zhong-Feng Zhang *

Contents

Table S1. Phenolic acids and flavonoids found in wild rice in previous reports. 1

Table S2. Physical properties of six macroporous resins. 6

Table S3. The ion transitions, optimized MS parameters, and linear relationships of the standards in UPLC-QqQ-MS/MS analysis. 7

Figure S1. The radical scavenging activities of DPPH (A) and ABTS (B), reducing power (C), total flavonoid content (TFC) (D), and total phenolic content (TPC) (E) of different solvent extracts of wild rice collected from Jingzhou. Different letters within the same figure mean statistical difference ($p < 0.05$). 9

Figure S2. Dynamic breakthrough curve of antioxidants on D101 resin column. 10

Figure S3. Structures of phenolic compounds identified in wild rice. 13

Figure S4. Base peak chromatograms of Fr. 1 (A) and Fr. 2 (B) in HPLC-LTQ-Orbitrap-MS^n analysis. 14

Figure S5. HPLC fingerprint chromatograms at 360 nm (A) and 280 nm (B) of the four fractions (Frs. 1–4) eluted from D101 resin column. The gradient solvent system consisting of A (methanol) and B (water containing 0.1% acetic acid, *v*/*v*) was as follows: 0–10 min, 5–10% A; 10–30 min, 10–15% A; 30–40 min, 15–20% A; 40–50 min, 20–25% A; 50–60 min, 25–35% A; 60–70 min, 35–90% A; 70–80 min, 90–60% A. 15

Table S1. Phenolic acids and flavonoids found in wild rice in previous reports.

Compound	Species	Collection Region of Sample	Identification Method	Reference
Phenolic acids and their derivatives				
Trans-ferulic acid	*Z. aquatica*	North-Ontario and Saskatchewan, Canada	HPLC	[1]
Cis-ferulic acid				
Ferulic acid	*Z. aquatica*	Dinorwic, Ontario, Canada and Houston, Texas, USA	HPLC-MS/MS	[2]
		Canada, Greece, Hungary, Cambodia	HPLC	[3]
Chlorogenic acid	*Z. aquatica*	Canada, Greece, Hungary, Cambodia	HPLC	[3]
Caffeic acid				
Gallic acid				
Ellagic acid				
o-Coumaric acid				
Protocatechuic acid ethyl ester				
Cinnamic acid				
Trans-*p*-coumaric acid	*Z. aquatica*	North-Ontario and Saskatchewan, Canada	HPLC	[1]
Cis-*p*-coumaric acid				
p-Coumaric acid	*Z. aquatica*	Dinorwic, Ontario, Canada and Houston, Texas, USA	HPLC-MS/MS	[2]
		Canada, Greece, Hungary, Cambodia	HPLC	[3]
Trans-sinapic acid	*Z. aquatica*	North-Ontario and Saskatchewan, Canada	HPLC	[1]
Sinapic acid	*Z. aquatica*	Dinorwic, Ontario, Canada and Houston, Texas, USA	HPLC-MS/MS	[2]
		Canada, Greece, Hungary, Cambodia	HPLC	[3]
p-Hydroxybenzoic acid	*Z. aquatica*	North-Ontario and Saskatchewan, Canada	HPLC	[1]
		Dinorwic, Ontario, Canada and Houston, Texas, USA	HPLC-MS/MS	[2]
		Canada, Greece, Hungary,	HPLC	[3]

		Cambodia		
Protocatechuic acid	*Z. aquatica*	North-Ontario and Saskatchewan, Canada	HPLC	[1]
		Canada, Greece, Hungary, Cambodia	HPLC	[3]
Vanillic acid	*Z. aquatica*	North-Ontario and Saskatchewan, Canada	HPLC	[1]
		Dinorwic, Ontario, Canada and Houston, Texas, USA	HPLC-MS/MS	[2]
		Canada, Greece, Hungary, Cambodia	HPLC	[3]
Syringic acid	*Z. aquatica*	North-Ontario and Saskatchewan, Canada	HPLC	[1]
		Dinorwic, Ontario, Canada and Houston, Texas, USA	HPLC-MS/MS	[2]
		Canada, Greece, Hungary, Cambodia	HPLC	[3]
p-Hydroxybenzaldehyde	*Z. aquatica*	North-Ontario and Saskatchewan, Canada	HPLC	[1]
		Dinorwic, Ontario, Canada and Houston, Texas, USA	HPLC-MS/MS	[2]
Protocatechuic aldehyde	*Z. aquatica*	North-Ontario and Saskatchewan, Canada	HPLC	[1]
Vanillin	*Z. aquatica*	North-Ontario and Saskatchewan, Canada	HPLC	[1]
		Dinorwic, Ontario, Canada and Houston, Texas, USA	HPLC-MS/MS	[2]
O-*β*-D-xylopyranosyl-(1→4)-*O*-[5-*O*-(*trans*-feruloyl)-*α*-L-arabinofuranosyl-(1→3)]-*O*-*β*-D-xylopyranosyl-(1→4)-D-xylopyranose	*Z. aquatica*	North-Ontario and Saskatchewan, Canada	MS, NMR	[1]
{[5-*O*-(*trans*feruloyl)][*O*-*β*-D-xylopyranosyl				

-(1→ 2)]-*O*-*α*-L-arabinofuranosyl-(1→ 3)}-*O*-*β*-D-xylopyranosyl-(1→4)-*D*-xylopyranose				
O-[5-*O*-(*trans*-feruloyl)-*α*-L-arabinofuranosyl]-(1→3)-*O*-*β*-D-xylopyranosyl-(1→ 4)-D-xylopyranose				
D-xylopyranosyl-(1 → 2)-[5-*O*-(*trans*-feruloyl)-L-arabinofuranose				
5-*O*-(*trans*-feruloyl)-L-arabinofuranose				
Cyclic form of 8–8-coupled dehydrodiferulic acid	*Z. aquatica*	obtained from a local German supplier	GC-MS, GC-FID	[4]
Noncyclic form of 8–8-coupled dehydrodiferulic acid	*Z. aquatica*	obtained from a local German supplier	GC-MS, GC-FID	[4]
		Dinorwic, Ontario, Canada and Houston, Texas, USA	HPLC-MS/MS	[2]
Cyclic form of 8–8-coupled dehydrodisinapic acid (thomasidioic acid)	*Z. aquatica*	obtained from a local German supplier	GC-MS, GC-FID	[4]
		Dinorwic, Ontario, Canada and Houston, Texas, USA	HPLC-MS/MS	[2]
Noncyclic form of 8–5-coupled dehydrodiferulic acid	*Z. aquatica*	obtained from a local German supplier	GC-MS, GC-FID	[4]
Noncyclic form of 8–8/7–*O*–7-coupled dehydrodiferulic acid				
Noncyclic form of 8–8-coupled	*Z. aquatica*	obtained from a local German supplier	GC-MS, GC-FID	[4]

dehydrodisinapic acid		Dinorwic, Ontario, Canada and Houston, Texas, USA	HPLC-MS/MS	[2]
8–*O*–4-coupled dehydrodiferulic acid	*Z. aquatica*	obtained from a local German supplier	GC-MS, GC-FID	[4]
		Dinorwic, Ontario, Canada and Houston, Texas, USA	HPLC-MS/MS	[2]
Cyclic form of 8–5-coupled dehydrodiferulic acid	*Z. aquatica*	obtained from a local German supplier	GC-MS, GC-FID	[4]
		Dinorwic, Ontario, Canada and Houston, Texas, USA	HPLC-MS/MS	[2]
5–5-Coupled dehydrodiferulic acid	*Z. aquatica*	obtained from a local German supplier	GC-MS, GC-FID	[4]
		Dinorwic, Ontario, Canada and Houston, Texas, USA	HPLC-MS/MS	[2]
Decarboxylated noncyclic form of 8–5-coupled dehydrodiferulic acid	*Z. aquatica*	obtained from a local German supplier	GC-MS, GC-FID	[4]
Flavonoids				
6,8-Di-*C*-glucosyl apigenin	*Z. palustris*, *Z. aquatica*	Wabigoon Lake, northwestern Ontario, Canada	HPLC-MS/MS	[5]
6-*C*-Glucosyl-8-*C*-arabinosyl apigenin				
6,8-Di-*C*-arabinosyl apigenin				
Procyanidin dimer				
Procyanidin trimer				
Procyanidin tetramer				
Procyanidin pentamer				
Catechin	*Z. palustris*, *Z. aquatica*	Wabigoon Lake, northwestern Ontario, Canada	HPLC-MS/MS	[5]
	Z. aquatica	Canada, Greece, Hungary,	HPLC	[3]

		Cambodia		
Epicatechin	*Z. palustris, Z. aquatica*	Wabigoon Lake, northwestern Ontario, Canada	HPLC-MS/MS	[5]
	Z. aquatica	Canada, Greece, Hungary, Cambodia	HPLC	[3]
Epigallocatechin	*Z. aquatica*	Canada, Greece, Hungary, Cambodia	HPLC	[3]
Rutin				
Quercetin				
Kaempferol				

Table S2. Physical properties of six macroporous resins.

Resin	Polarity	Particle Size (mm)	Surface Area (m^2/g)	Average Pore Diameter (Å)
HPD600	Polar	0.30 – 1.20	550 – 600	80
NKA-9	Polar	0.30 – 1.25	250 – 290	155 – 165
AB-8	Weak-polar	0.03 – 1.25	480 – 520	130 – 140
X-5	Non-polar	0.30 – 1.25	500 – 600	290 – 300
D101	Non-polar	0.30 – 1.25	550 – 600	90 – 100
HPD300	Non-polar	0.30 – 1.20	800 – 870	50 – 55

Table S3. The ion transitions, optimized MS parameters, and linear relationships of standards in UPLC-QqQ-MS/MS analysis.

Compound	Parent Ion (*m*/*z*)	Product Ion (*m*/*z*)	Collision Energy (V)	t_R[a] (min)	Calibration Curve[b]	r^2	LOD[c] (ng/mL)	LOQ[d] (ng/mL)
p-Hydroxybenzaldehyde	121.138	92.224	25	2.91	$y = 8.2754\times10^4x + 5.1399\times10^4$	0.9991	0.992	3.298
		120.306	20					
p-Hydroxybenzoic acid	137.116	65.238	31	2.03	$y = 2.3214\times10^5x - 3.1002\times10^5$	0.9982	4.691	14.539
		75.046	32					
		93.220	17					
Vanillin	151.120	51.600	50	4.05	$y = 6.5972\times10^5x - 5.8837\times105$	0.9981	6.936	20.013
		92.239	23					
		107.962	25					
		136.138	16					
Protocatechuic acid	153.095	91.099	27	1.01	$y = 7.4267\times10^4x - 9.8661\times10^3$	0.9995	0.770	2.566
		108.084	26					
		109.179	17					
p-Coumaric acid	163.080	93.221	37	3.18	$y = 4.4936\times10^5x - 6.9351\times10^5$	0.9999	3.393	10.643
		117.187	37					
		119.199	18					
o-Coumaric acid	163.095	93.047	36	6.11	$y = 2.8759\times10^5x + 3.9233\times10^5$	0.9988	5.075	16.251
		117.171	25					
		119.185	15					
Vanillic acid	167.042	91.078	22	2.84	$y = 2.0862\times10^5x - 3.1034\times10^5$	0.9993	6.294	19.648
		108.118	19					
		123.106	13					
		152.027	16					
Gallic acid	169.081	79.288	24	0.61	$y = 7.3772\times10^4x - 3.4494\times10^4$	0.9994	2.589	8.630
		97.217	20					
		124.132	27					
		125.176	15					
Protocatechuic acid ethyl ester	181.038	108.487	23	7.39	$y = 5.9352\times10^4x + 2.5769\times10^6$	0.9992	0.147	0.491
		151.949	16					
		153.005	16					
Ferulic acid	193.081	133.097	30	5.16	$y = 5.8092\times10^4x - 4.0636\times10^4$	0.9982	3.321	11.088
		134.166	18					
		149.184	13					
		178.117	14					
Syringic acid	197.084	95.222	35	3.36	$y = 2.6176\times10^5x - 4.1087\times10^5$	0.9996	6.213	20.712
		123.166	25					

		167.117	21					
		182.117	16					
Sinapic acid	223.065	149.105	23	6.31	$y = 3.1595\times10^5x$ -8.3289×10^4	0.9979	4.868	16.226
		164.141	18					
		193.081	25					
		208.100	17					
Catechin	289.041	203.256	21	2.97	$y = 1.3143\times10^4x$ -9.6578×10^3	0.9995	8.395	27.984
		245.129	17					
Epicatechin	289.050	203.210	23	3.56	$y = 2.1011\times10^4x$ -1.3420×10^4	0.9991	6.787	22.067
		245.137	18					
Quercetin	301.000	107.184	33	7.92	$y = 1.7855\times10^5x$ -5.8558×10^4	0.9997	0.297	0.991
		121.176	30					
		151.074	24					
		179.060	20					
Epigallocatechin	305.033	125.138	27	2.35	$y = 3.9510\times10^4x$ -1.5239×10^4	0.9990	2.744	9.148
		165.082	20					
		179.110	18					
		219.098	19					
Procyanidin B1	577.128	289.088	26	2.60	$y = 9.1478\times10^4x$ -3.5465×10^4	0.9997	2.720	9.068
		407.009	23					
		425.148	18					
Procyanidin B2	577.137	289.010	26	2.85	$y = 1.1698\times10^5x$ -8.5724×10^4	0.9989	1.894	6.332
		407.002	24					
		425.159	19					
Procyanidin B3	577.112	289.017	28	3.63	$y = 1.0529\times10^5x$ -4.0134×10^4	0.9998	2.215	7.365
		407.021	24					
		425.098	17					
Rutin	609.122	151.051	52	6.79	$y = 2.5348\times10^6x$ $+2.4363\times10^6$	0.9989	0.012	0.039
		255.055	58					
		271.093	57					
		300.142	40					
Procyanidin C1	865.147	386.970	38	7.87	$y = 8.0104\times10^4x$ $+1.5888\times10^4$	0.9999	0.416	1.385
		524.985	29					
		695.291	26					
		713.302	29					

[a] t_R, retention time. [b] The calibration curve was constructed by plotting the peak area versus the concentration of each standard. [c] LOD, limits of detection. [d] LOQ, limits of quantification. Precision of the data obtained from repeated experiments (RSD%) < 7%.

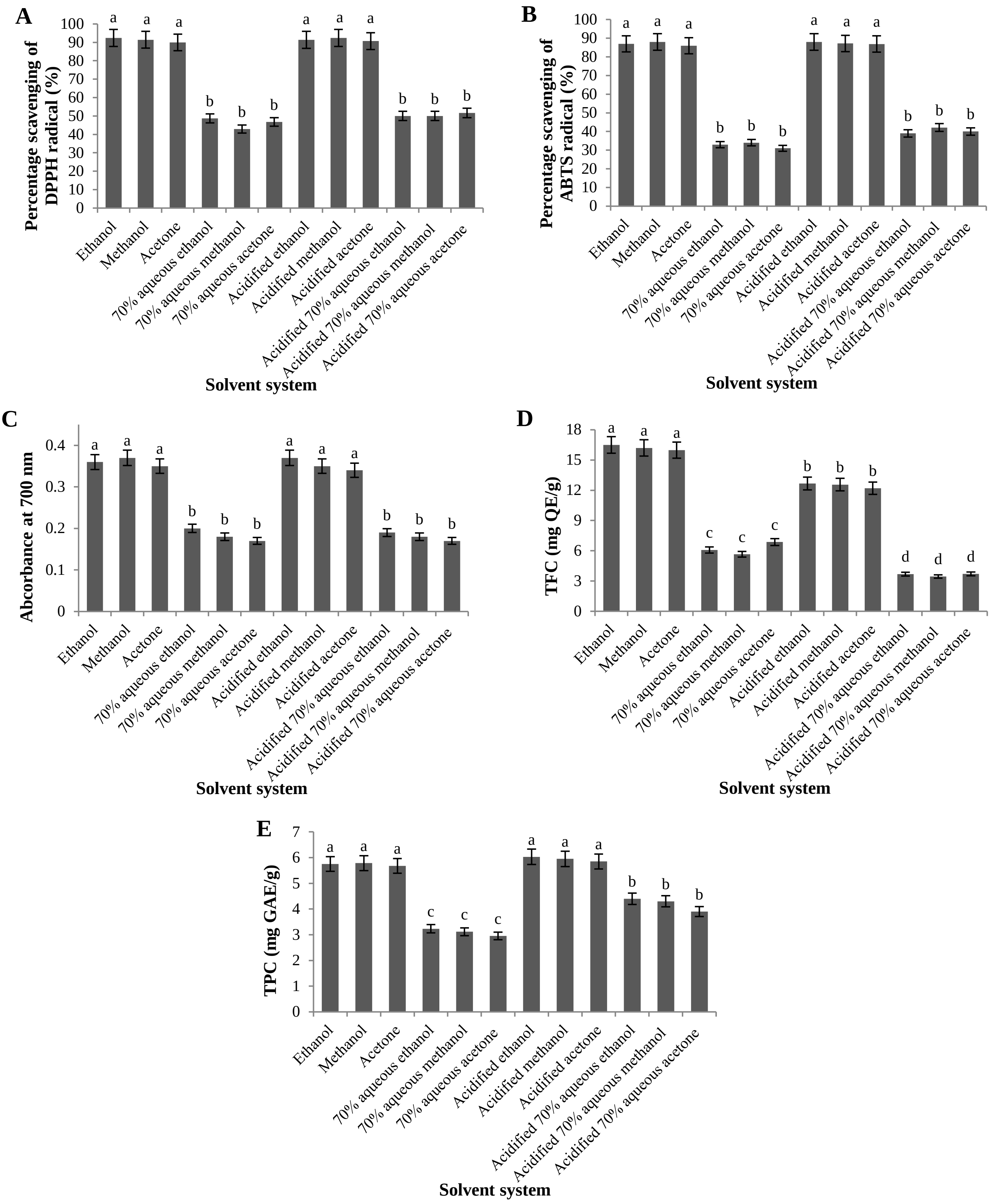

Figure S1. The radical scavenging activities of DPPH (**A**) and ABTS (**B**), reducing power (**C**), total flavonoid content (TFC) (**D**), and total phenolic content (TPC) (**E**) of different solvent extracts of wild rice collected from Jingzhou. Different letters within the same figure mean statistical difference ($p < 0.05$).

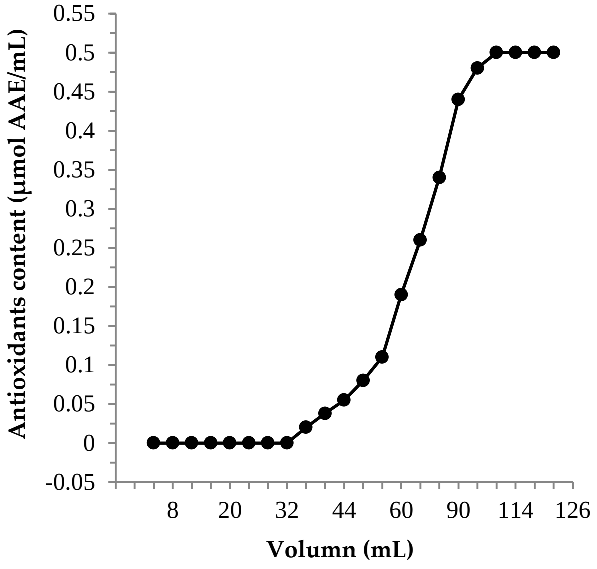

Figure S2. Dynamic breakthrough curve of antioxidants on D101 resin column.

Phenolic acids and their derivatives

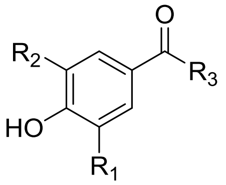

A1: R_1=OH, R_2=OH, R_3=OH
A2: R_1=H, R_2=OH, R_3=OH
A3: R_1=H, R_2=H, R_3=OH
A4: R_1=H, R_2=OMe, R_3=OH
A5: R_1=H, R_2=H, R_3=H
A6: R_1=OMe, R_2=OMe, R_3=OH
A7: R_1=H, R_2=OMe, R_3=H
A8: R_1=H, R_2=OH, R_3=OEt

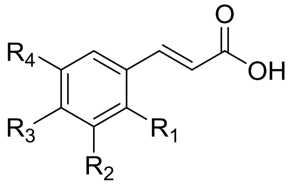

A9: R_1=H, R_2=H, R_3=OH, R_4=H
A10: R_1=OH, R_2=H, R_3=H, R_4=H
A11: R_1=H, R_2=OMe, R_3=OH, R_4=H
A12: R_1=H, R_2=OMe, R_3=OH, R_4=OMe
B21: R_1=H, R_2=OMe, R_3=OMe, R_4=OMe

Procyanidins

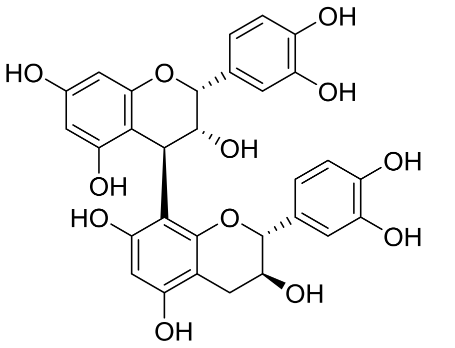

B1

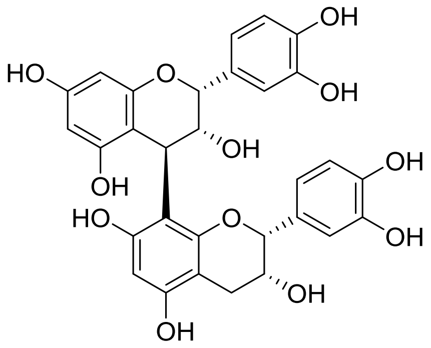

B2

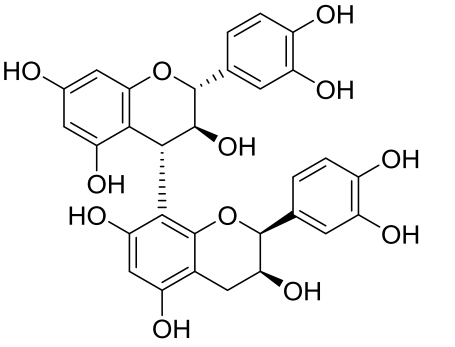

B3

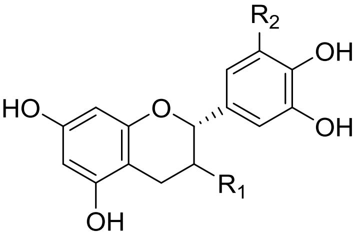

B4: R_1= OH, R_2=OH
B5: R_1= OH, R_2=H
B6: R_1= OH, R_2=H

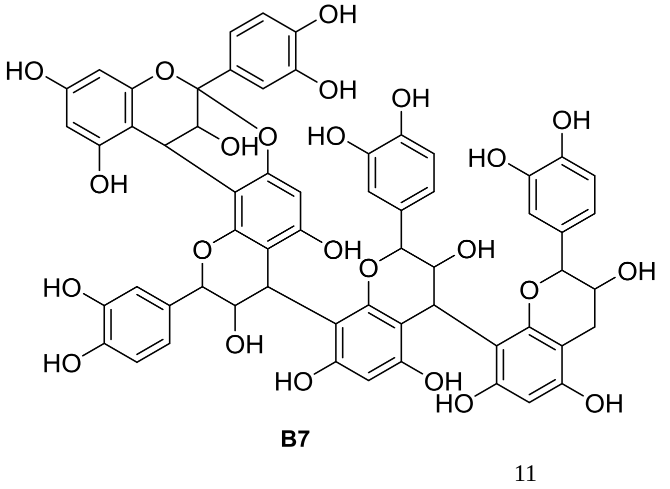

B7

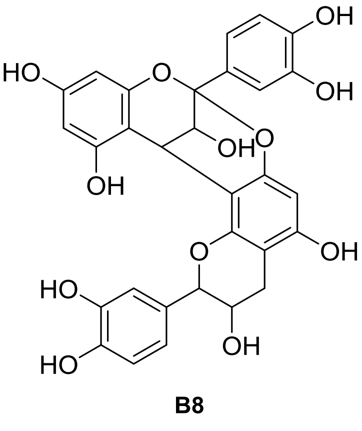

B8

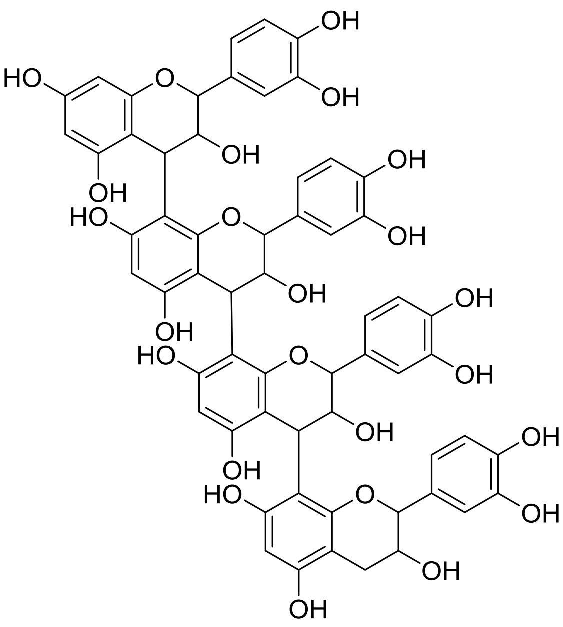

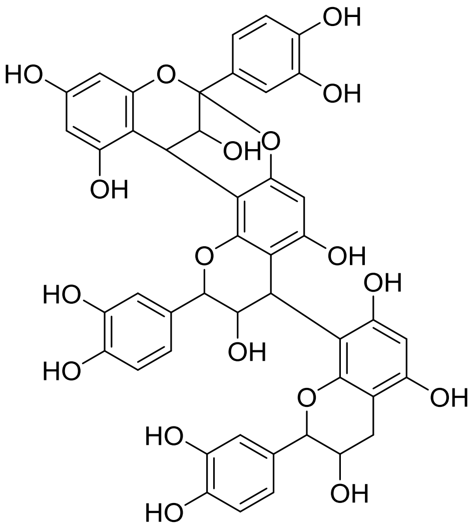

B11

B13

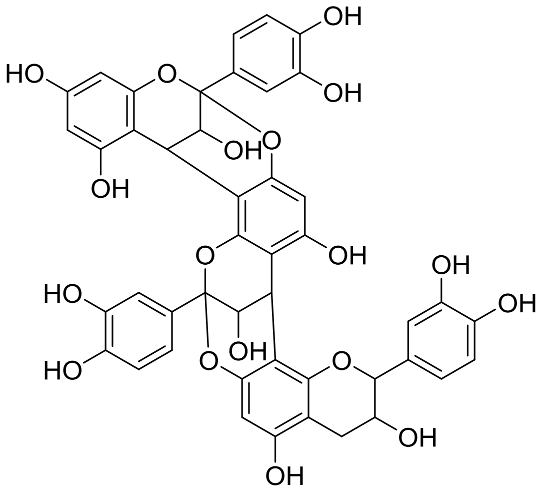

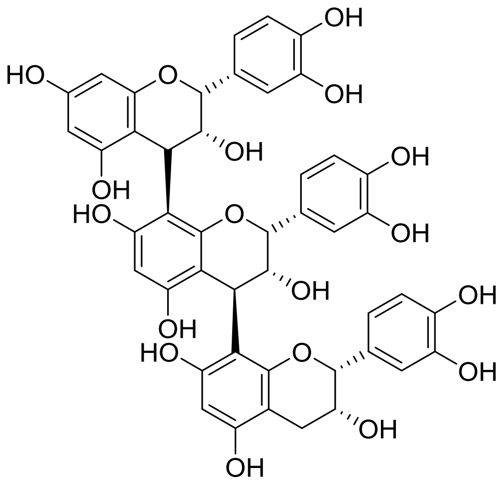

B18

B19

Flavonoid glycosides

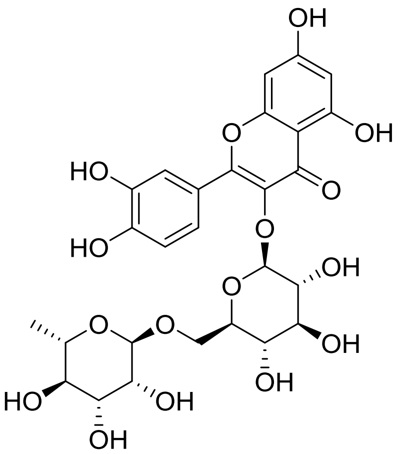

B9

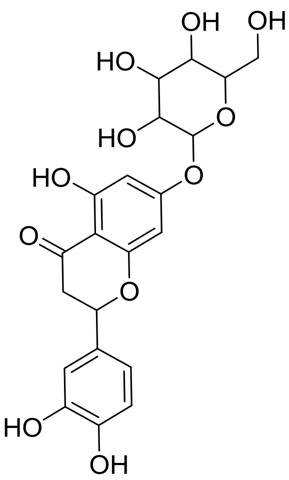

B10

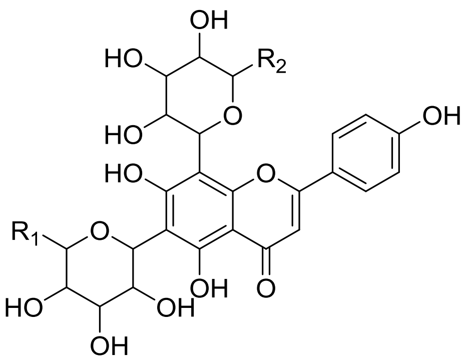

B12: $R_1=CH_2OH$, $R_2=CH_2OH$
B15: $R_1=CH_2OH$, $R_2=H$
B16: $R_1=H$, $R_2=CH_2OH$
B17: $R_1=H$, $R_2=H$

Others

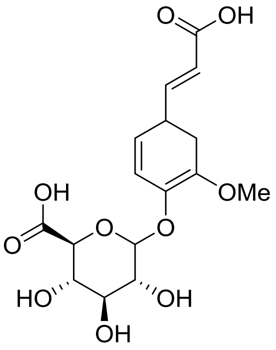

B14

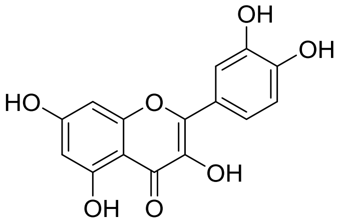

B20

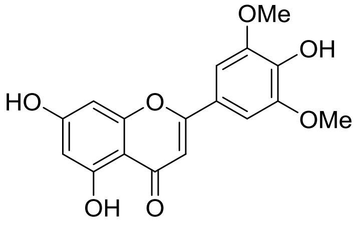

B22

Figure S3. Structures of phenolic compounds identified in wild rice.

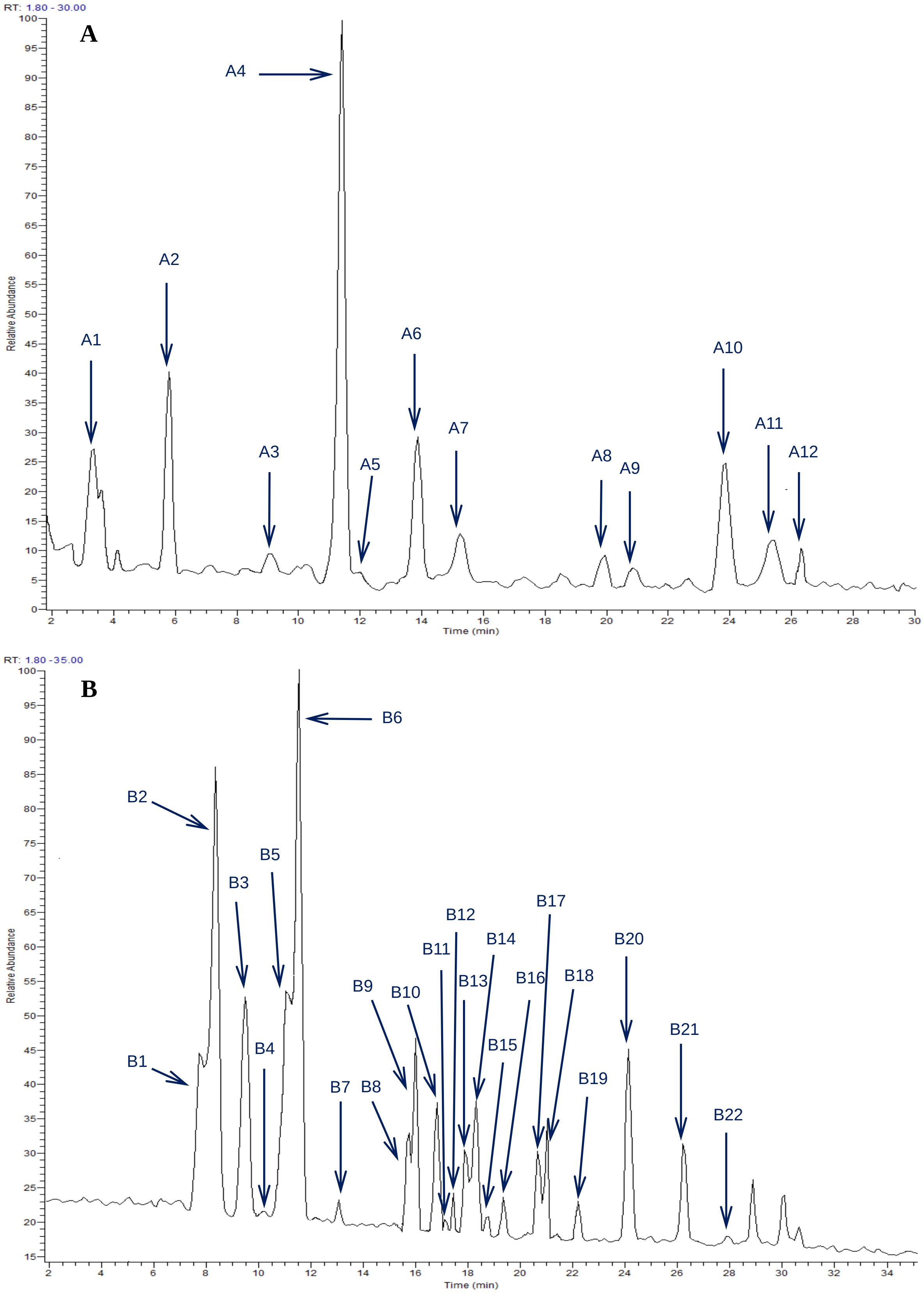

Figure S4. Base peak chromatograms of Fr. 1 (**A**) and Fr. 2 (**B**) in HPLC-LTQ-Orbitrap-MS^n analysis.

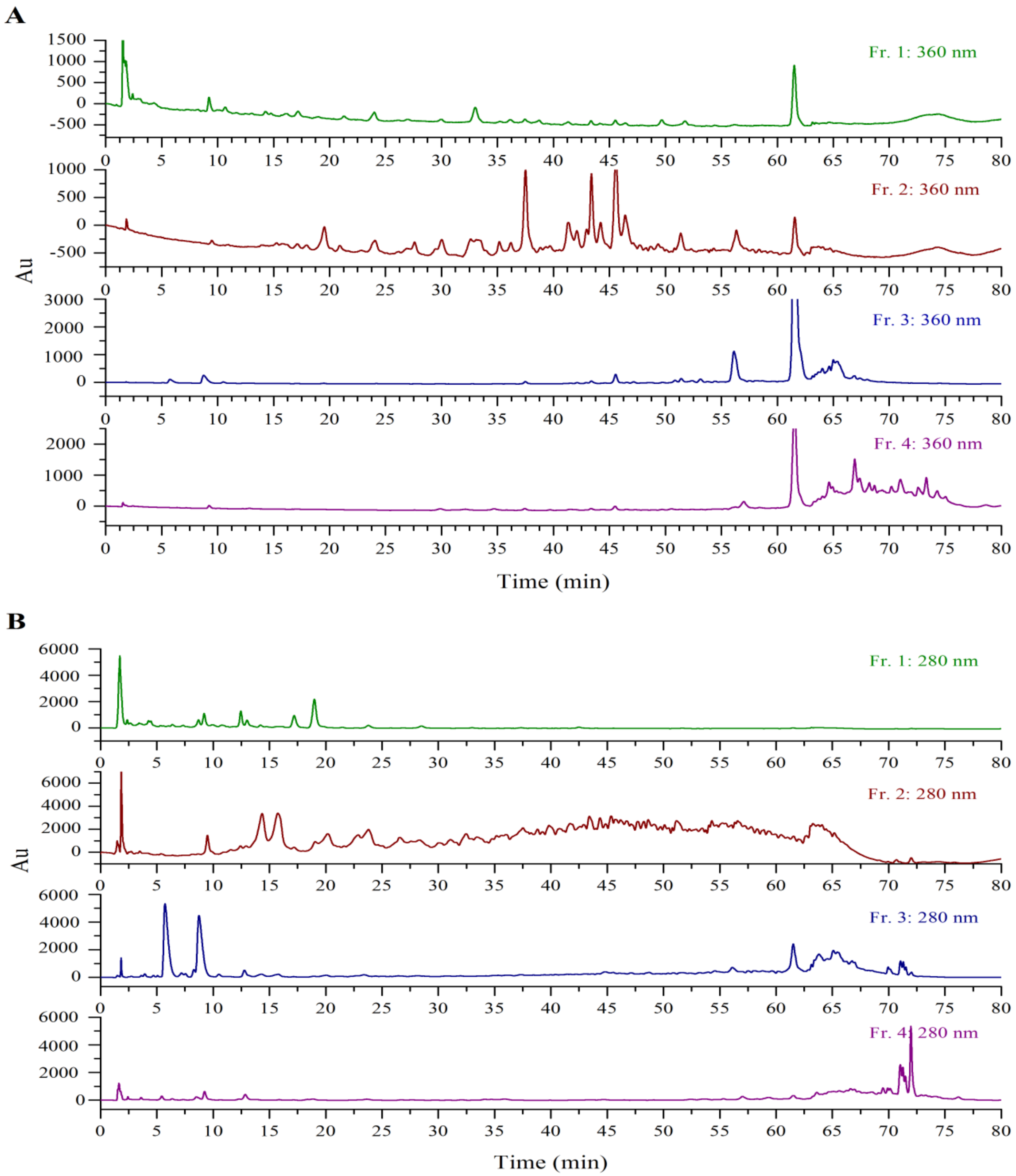

Figure S5. HPLC fingerprint chromatograms at 360 nm (**A**) and 280 nm (**B**) of the four fractions (Frs. 1–4) eluted from D101 resin column. The gradient solvent system consisting of A (methanol) and B (water containing 0.1% acetic acid, *v*/*v*) was as follows: 0–10 min, 5–10% A; 10–30 min, 10–15% A; 30–40 min, 15–20% A; 40–50 min, 20–25% A; 50–60 min, 25–35% A; 60–70 min, 35–90% A; 70–80 min, 90–60% A.

References

1. Bunzel, M.; Allerdings E.; Sinwell, V.; Ralph J.; Steinhart H. Cell wall hydroxycinnamates in wild rice (*Zizania aquatica* L.) insoluble dietary fibre. *Eur. Food. Res. Technol.* **2002**, *214*, 482–488.
2. Qiu, Y.; Liu Q.; Beta, T. Antioxidant properties of commercial wild rice and analysis of soluble and insoluble phenolic acids. *Food Chem.* **2010**, *121*, 140−147.
3. Sumczynski D.; Kotásková E.; Orsavová J.; Valášek P. Contribution of individual phenolics to antioxidant activity and *in vitro* digestibility of wild rices (*Zizania aquatica* L.). *Food Chem.* **2017**, *218*, 107−115.
4. Bunzel, M.; Ralph, J.; Kim, H.; Lu, F.; Ralph, S. A.; Marita, J. M.; Hatfield, R. D.; Steinhart, H. Sinapate dehydrodimers and sinapate−ferulate heterodimers in cereal dietary fiber. *J. Agric. Food Chem.* **2003**, *51*, 1427−1434.
5. Qiu, Y.; Liu Q.; Beta, T. Antioxidant activity of commercial wild rice and identification of flavonoid compounds in active fractions. *J. Agric. Food Chem*. **2009**, *57*, 7543−7551.
